# Supplementary material for: Sequential modification of bacterial chemoreceptors is key for achieving both accurate adaptation and high gain
Source: Nat Commun. 2020 Jun 8;11:2875. doi: 10.1038/s41467-020-16644-4 (PMC7280522; doi:10.1038/s41467-020-16644-4)
Supplement: Supplementary file 1 — Supplementary Information [file 41467_2020_16644_MOESM1_ESM.pdf]

Supplementary Information

Sequential modification of bacterial chemoreceptors is key for achieving accurate adaptation and high gain simultaneously

Bernardo A. Mello, Anderson B. Beserra, and Yuhai Tu

SUPPLEMENTARY METHODS

Monte-Carlo simulations. The Ising model [57] was used to simulate a cluster of single type receptors arranged in a 64×64 square lattice. The state of the receptor i is defined by its activity (a_i), occupancy (l_i), and modification state (m_i for sequential modification or $\boldsymbol{\mu}_i$ for random modification). Because the variables a_i and l_i are considered to be in quasi-static equilibrium, we use a MC simulation with the probability of the states following the Boltzmann distribution with the Hamiltonian

$$E_i = (\alpha_0 + a_i\alpha)(m_i - m_0) + l_i \log \frac{K_{a_i}}{[L]} + C(a_i - 0.5) \sum_{i'}^{\substack{\text{neigh} \\ \text{of i}}} (a_{i'} - 0.5) \ . \tag{1}$$

The dissociation constants of inactive and active receptors are, respectively, $K_\mathrm{I} = 18\,\mu\mathrm{M}$ and $K_\mathrm{A} = 3000\,\mu\mathrm{M}$ from [70].

Monte-Carlo simulation is also used for the out-of-equilibrium modification dynamics [72]. Arrows pointing to the right in Fig. 1(b) represent the addition of methyl groups, which occurs only on active receptors ($a = 1$), with transition probability rate k^+ for black arrows and ηk^+ for gray arrows. Arrows pointing to the left in Fig. 1(b) represent the removal of methyl groups, which occurs only on inactive receptors ($a = 0$), with transition probability rate k^- for black arrows and ηk^- for gray arrows.

The quasi-static equilibrium of a and l is obtained by the rate of transition between the states of these variables been, on average, at least 10 time faster the the rate of transitions between the mehtylation states $\boldsymbol{\mu}$, per receptor.

The following pseudo-code describes the Monte-Carlo procedure in a $N \times N$ lattice of receptors:

```
function site_energy(a, l, i, j) :
    * Returns the energy with the present receptors lattice configuration,
    * except for a_ij = a and l_ij = l.

t ← 0
while t < t_Final :
    for k from 1 to M :
        i ← int_random([1..N])
        j ← int_random([1..N])
        m ← int_random([1..M])
        if a_ij = 0 and μ_ij,m = 0 :
            if float_random([0..1]) < (μ_ij,m−1 + η(1 − μ_ij,m−1)) k⁺Δt :
                μ_ij,m ← 1
        if a_ij = 1 and μ_ij,m = 1 :
            if float_random([0..1]) < ((1 − μ_ij,m+1) + ημ_ij,m+1) k⁻Δt :
                μ_ij,m ← 0
    for k from 1 to 200 × Δt :
        i ← int_random([1..N])
        j ← int_random([1..N])
        E_00 ← exp(−site_energy(0, 0, i, j))
        E_01 ← exp(−site_energy(0, 1, i, j))
        E_10 ← exp(−site_energy(1, 0, i, j))
        E_11 ← exp(−site_energy(1, 1, i, j))
        r = float_random([0..E_00 + E_01 + E_10 + E_11])
        if r < E_00 :
            a_ij ← 0
            l_ij ← 0
        else :
            r ← r − E_00
```

if $r < E_{01}$:

$a_{ij} \leftarrow 0$

$l_{ij} \leftarrow 1$

else :

$r \leftarrow r - E_{01}$

if $r < E_{10}$:

$a_{ij} \leftarrow 1$

$l_{ij} \leftarrow 0$

else :

$a_{ij} \leftarrow 1$

$l_{ij} \leftarrow 1$

$t \leftarrow t + \Delta t$

Details of Computing Γ and ξ. The amplification gain (Γ) and the adaptation error (ξ) are obtained by MC simulation. The simulation begins with the lowest background concentration 10^{-1} µM. After the system is adapted to its current background, the value of $[L]$ is increased by a small fraction $\delta[L] = 0.1[L]$ and the fast response of the system, $\delta\langle a\rangle$, is measured as the difference between the activity immediately before and immediately after the stimulus change, as given by Eq. (15). At the $i-$th step, the background concentration is increased by a factor $\sqrt{10}$ from its current level $[L]_i$ to the next level $[L]_{i+1} = \sqrt{10}[L]_i$ and the process of waiting for adaptation and measuring the system response to a small fractional change $\delta[L]_{i+1} = 0.1[L]_{i+1}$ is repeated. We continue this process until the highest background concentration 10^6 µM is reached. These steps are then performed in the opposite direction, i.e., with the background concentration decreasing from 10^6 µM to 1 µM by a factor $\sqrt{10}$ for every step and the responses to $\delta[L] = -0.1[L]$ were computed for each background levels. We stress that $\delta[L]_i = 0.1[L]_i$ is not the difference $[L]_{i+1} - [L]_i$, but a much smaller change used to evaluate the system's linear response.

In our simulations, two signal gains, $g^+([L])$ and $g^-([L])$, can be computed for the responses to a small fractional increase ($\delta[L] = 0.1[L]$) and a small fractional decrease ($\delta[L] = -0.1[L]$) of the input, respectively. Supplementary Fig. 1 shows $g^+([L])$ and

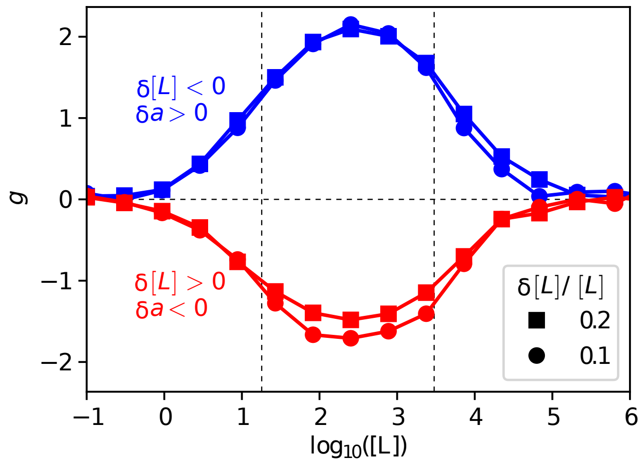

FIG. 1. Response gains as a function of $[L]$. The response gain defined by Eqs. (15)&(16) in the main text as a function of the log of the ligand concentration $log_{10}([L])$. The blue lines (symbols) are for $g^+([L])$ the response gain to a fractional increase of signal $\delta[L]/[L] = 0.1$(circle), 0.2(square). The red lines (symbols) are for $-g^-([L])$ with g^- computed with a fractional decrease of signal $\delta[L]/[L] = -0.1$(circle), -0.2(square). The vertical dashed lines are the values of $[L]$ corresponding to K_{I} and K_{A}. The unit of $[L]$ is in µM. The full range of background concentrations studied here is from $10^{-1}\mu M$ to $10^{6}\mu M$.

$-g^-([L])$ as a function of $[L]$. The results do not seem to vary significantly for $\delta[L] = 0.1[L]$ and $\delta[L] = 0.2[L]$ as shown in Supplementary Fig. 1, we use $\delta[L]/[L] = 0.1$ in our study. Due to the small but finite values of $\delta[L]$, $g^+([L])$ and $g^-([L])$ can be slightly different from each other, and we approximate $g([L]) \approx [g^+([L]) + g^-([L])]/2$ as their average. The overall gain Γ defined in Eq. 17 in the main text can thus be numerically calculated as

$$\Gamma \approx \sum_i \frac{g^+([L]_i) + g^-([L]_i)}{2} \log_{10} \frac{[L]_{i+1}}{[L]_i} \,. \tag{2}$$

Details of the simulations for comparison with the experiments. The experimental value $2.3 < \xi^{-1} < 3.5$ was obtained with data from [74] where the adapted activity was measured with maximum reliable MeAsp concentration 5 mM. To have a comparable result we simulated the value of ξ with $[L]_{max} = 5$ mM and produced the dependence of $\xi^{-1} \times \eta$ shown in Supplementary Fig. 2. With the help of that plot we found the value of η corresponding to each value of ξ^{-1}, obtaining $0.05 \leq \eta \leq 0.13$.

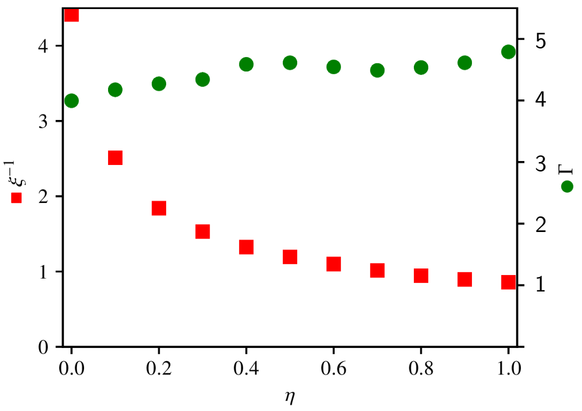

FIG. 2. The dependence of Γ and ξ^{-1} on η. Here, ξ^{-1} is calculated using $[L]_{\max} = 5$ mM, the largest reliable experimental value available for MeAsp in [74]. All other parameters used are the same as in Fig.3a in the main text.

SUPPLEMENTARY NOTE 1

Accurate adaptation with sequential modification. Previous theoretical analysis showed that perfect adaptation requires three conditions [57, 58]:

1. Modification and reverse modification rates depend only on the receptor activity, not on the m-state or ligand binding state.

2. Modification is sequential, implying in one single m-state for each m-level ($\eta = 0$).

3. Modification of $m = M$ receptors ($k_M^+ P_M$) and the reverse modification of $m = 0$ receptors ($k_0^- P_0$) must be zero in Supplementary Eq. (9).

Condition 3 depends on the boundary modification states, $m = 0$ and $m = M$. Sequential modification can exhibit very precise, though imperfect, adaptation in a broad range of stimulus concentration. The size of the adaptation error depends on the extent of the violation of condition 3.

There are two ways of complying with condition 3. One is by having no receptor at states $m = 0$ and $m = M$, $P_0 = P_M = 0$, as is the case of $m = 0$ and $[L] = 10^5$ μM and of $m = 4$ with $[L] = 1$ μM and 10^1 μM in Supplementary Fig. 3. Another way is by having receptors in these states, but only inactive ones in $m = 0$ and only active ones in $m = 4$, as is the case of $m = 0$ with $[L] = 1$ μM and $m = 4$ with $[L] = 10^5$ μM in Supplementary

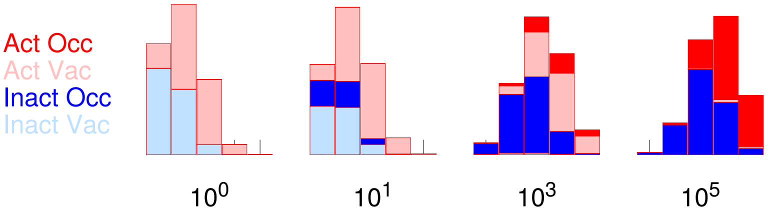

FIG. 3. Steady state distribution of receptor ligand occupancy and kinase activity in different methylation levels for the sequential modification model ($\eta = 0$). The numbers below the panels are the attractant concentration in μM. The horizontal axis is the m-level ($m \in [0, 4]$). The different color represents the fraction of receptors in one of the four states – active or inactive and vacant or occupied (by the ligand). The columns (bars) from left to right correspond to different methylation levels from $m = 0$ to $m = 4$.

Fig. 3. The other configurations of $m = 0$ and $m = 4$ in Supplementary Fig. 3 lead to imperfect adaption.

The focus of our current study is on the slow population dynamics of receptor methylation/demethylation described by the transition rate $k_{\mu,\mu'}$ from state μ to μ'. The probability $P_\mu(,t)$ of each of the 2^M states is governed by the master equation

$$\frac{dP_\mu}{dt} = \sum_{\mu'} k_{\mu',\mu} P_\mu - P_\mu \sum_{\mu'} k_{\mu,\mu'} \ . \tag{3}$$

The modification dynamics are determined by the specific choices of $k_{\mu,\mu'}$, which is a function of the mean activity of the initial state (Eq. (1)).

For purely sequential dynamics, μ can be replaced by m in Supplementary Eq. (3) and the number of equations is reduced to $M + 1$,

$$\frac{dP_m}{dt} = \sum_{m} k_{m',m} P_m - P_m \sum_{m'} k_{m,m'} \ . \tag{4}$$

Since transitions are only allowed between states with $m' = m \pm 1$, the above equation may be written as

$$\frac{dP_m}{dt} = k^+_{m-1} P_{m-1} + k^-_{m+1} P_{m+1} - (k^+_m + k^-_m) P_m \quad \text{for } 0 < m < M, \tag{5}$$

$$\frac{dP_0}{dt} = -k^+_0 P_0 + k^-_1 P_1 \ , \tag{6}$$

$$\frac{dP_M}{dt} = -k^-_M P_M + k^+_{M-1} P_{M-1} \ . \tag{7}$$

By substituting the above expressions and Eq. (1) in the time derivative of Eq. (12), we arrive to the dynamic equation of mean methylation for sequential modification ($\eta = 0$)

$$\frac{d\langle m\rangle}{dt} = k^+(1-\langle a\rangle) - k^-\langle a\rangle + \epsilon \tag{8}$$

with

$$\epsilon = -k^+(1-\langle a\rangle_M)P_M + k^-\langle a\rangle_0 P_0 \ . \tag{9}$$

Strict observation of the three conditions of Accurate adaptation with sequential modification in Supplementary Note 1 would result in the adapted activity being a horizontal straight line in Fig. 2(b). In particular, condition 3 leads to the boundary error $\epsilon = 0$ and by making

$$\left.\frac{d\langle m\rangle}{dt}\right|_{\langle m\rangle^{\mathcal{A}},\langle a\rangle^{\mathcal{A}}} = 0 \tag{10}$$

in Supplmentary Equation (8), we have

$$\langle a\rangle^{\mathcal{A}} = \frac{k^+}{k^+ + k^-} \ , \tag{11}$$

which is independent of $[L]$, i.e., perfect adaptation.

The master equation of P_m for $\eta = 1$ is obtained by replacing Supplementary Eq. (3) in the time derivative of Eq. (11). Since this dynamics is controlled by the average activity level of each state μ, which depends only on the activity level, i.e., $\langle a\rangle_{\mu} = \langle a\rangle_{m=\|\mu\|}$, we can group together the states in the same methylation level, multiplied by the number of sites available for methylation or demethylation,

$$\begin{aligned}\frac{dP_m}{dt} =&(M+1-m)k^+_{m-1}P_{m-1} + (m+1)k^-_{m+1}P_{m+1} \\ &- [(M-m)k^+_m + mk^-_m]P_m \quad \text{for } 0 < m < M,\end{aligned} \tag{12}$$

$$\frac{dP_0}{dt} =k^-_1 P_1 - Mk^+_0 P_0 \ \ , \tag{13}$$

$$\frac{dP_M}{dt} =k^+_{M-1}P_{M-1} - Mk^-_M P_M. \tag{14}$$

By substituting the above expressions and Eq. (1) in the time derivative of Eq. (12), we arrive to the dynamic equation of mean methylation for sequential modification ($\eta = 1$)

$$\frac{d\langle m\rangle}{dt} = k^+\langle(M-m)(1-\langle a\rangle_m)\rangle - k^-\langle m\langle a\rangle_m\rangle \ , \tag{15}$$

$$= k^+(M - \langle m\rangle - M\langle a\rangle + \langle m\langle a\rangle_m\rangle) - k^-\langle m\langle a\rangle_m\rangle \ . \tag{16}$$

The dependence of the above expression on the covariance of m and a, through $\langle m\langle a\rangle_m\rangle$, is eliminated in the particular case $k^+ = k^-$, for which

$$\frac{d\langle m\rangle}{dt} = k^+(M - \langle m\rangle - M\langle a\rangle) \ . \quad (17)$$

By imposing Supplementary Eq. (10) in this particular case, we obtain the expression of the adapted activity as a function of the adapted methylation level,

$$\langle a\rangle^{\mathcal{A}}([L]) = \frac{M - \langle m\rangle^{\mathcal{A}}([L])}{M} \ . \quad (18)$$

The right hand side of the above expression is not a constant, but decreases linearly with $\langle m\rangle^{\mathcal{A}}([L])$, that depends on the stimulus concentration. This is the cause of the partial adaptation with random modification.

The dependence of Γ and ξ^{-1} on α. The parameter α is the scaling factor of m in the Hamiltonian, Supplementary Eq. (1). Therefore, if m were a real number, increasing $|\alpha|$ would not only bring the curves of constant $[L]$ in Fig. 2a,b close to each other, but would also make them steeper by the same amount, resulting in the same change in $\langle a\rangle$ for a given change from $[L]$ to $[L]'$. Consequently, changing $|\alpha|$ would alter the adaptation precision without affecting the response gain. This is the case of the MWC model.

However, as shown in Supplementary Fig. 4, in the Ising-type model studied here, the precision is enhanced by increasing $|\alpha|$, with the collateral effect of decreasing the response gain. This difference is caused by the assumption that m is a real number in the MWC model as m is treated as an average methylation level over all the receptors in the all-or-none MWC cluster. The MC simulation of the Ising model properly define m as an integer number for each receptor. If $\langle m\rangle$ is not approximately integer, large values of $|\alpha|$ result in receptor population formed mostly of two levels of m, one that is very active and other that is very inactive. Their activity are minimally affected by changes of $[L]$. Because of it, increasing $|\alpha|$ reduces the response gain in the Ising model.

In the purely sequential case ($\eta = 0$), from Eq. (4), there is a small adaptation error ϵ proportional to the probability of receptors in the extreme (boundary) methylation state $m = M$ (or $m = 0$). For $[L] \gg K_{\mathrm{A}}$, P_m peaks at $\langle m\rangle^{\mathcal{A}}_\infty \approx m_0 + |\alpha|^{-1}\ln(K_{\mathrm{A}}/K_{\mathrm{I}})$ and decays exponentially for $m > \langle m\rangle^{\mathcal{A}}_\infty$ as $\exp[-(m - \langle m\rangle^{\mathcal{A}}_\infty)/\sigma_m]$ with $\sigma_m = b/|\alpha|$ where b is an order 1 constant. Therefore, the adaptation error for sequential modification follows:

$$\xi(\eta = 0) \approx c_1 \times \exp[-b^{-1}|\alpha|(M - m_0)] + \xi_0 \ , \quad (19)$$

where c_1 is a constant and ξ_0 is the error from the $m = 0$ state. Eq.(19) shows that ξ decreases exponentially with $|\alpha|$ before saturating to ξ_0.

In the case of random modification ($\eta = 1$), a much larger adaptation error occurs because the adaptation line (solid line) in Fig. 2(b) is tilted across the whole range of methylation level $0 \leq m \leq M$. The slope of the adapted activity dependence on the average methylation level $\langle m \rangle^{\mathcal{A}}$ is $\sim 1/M$ and the change in $\langle m \rangle^{\mathcal{A}}$ for the full stimulus range is $\Delta m \sim \ln(K_{\mathrm{A}}/K_{\mathrm{I}})/|\alpha|$, from which we can estimate the adaptation error:

$$\xi(\eta = 1) \approx \frac{\Delta m}{M} \approx \frac{\ln(K_{\mathrm{A}}/K_{\mathrm{I}})}{M}|\alpha|^{-1} , \tag{20}$$

which only decreases with $|\alpha|$ algebraically.

Results from direct MC simulations, as shown in Supplementary Fig. 4(a), confirmed the analytical expressions of ξ for the sequential ($\eta = 0$), Supplementary Eq. (19), and the random ($\eta = 1$), Supplementary Eq. (20), cases. Taken together, our analysis clearly shows that sequential modification enhances the adaptation accuracy much more efficiently than random modification. We also studied the gain Γ in our simulations. As shown in Supplementary Fig. 4(b), the dependence of Γ on α does not show much difference between sequential and random modification.

Enzymatic description of CheR/receptor interaction. Without knowing the details of the enzymatic reaction, the reaction between the methyltransferase cheR (enzyme E) and the methylation sites (substrate S) is assumed to follow a Michaelis Menten mechanism,

$$[E] + [S] \underset{k_{\mathrm{off}}}{\overset{k_{\mathrm{on}}}{\rightleftharpoons}} [SE] \xrightarrow{k_{\mathrm{cat}}} [S^*] + [E] . \tag{21}$$

If the methylation process is random, i.e., CheR can methylate each of the available methylation sites randomly, the substrate concentration becomes effectively $[S] = (M - m)[T]$ where $[T]$ is the receptor concentration. Even with this random methylation scheme, this reaction can result in a methylation rate independent of the methylation level under certain conditions. In particular, by assuming the rate limiting step is the catalytic reaction and the Michaelis-Mentum constant $K_{\mathrm{M}}(\approx k_{off}/k_{on})$ is small (relative to the substrate and/or enzyme concentrations),

$$k_{\mathrm{on}}([S]_0 + [E]_0) \gg k_{\mathrm{off}} \gg k_{\mathrm{cat}} , \tag{22}$$

with $[S]_0 = [S] + [SE]$ and $[E]_0 = [E] + [SE]$, it can be demonstrated, in the quasi steady

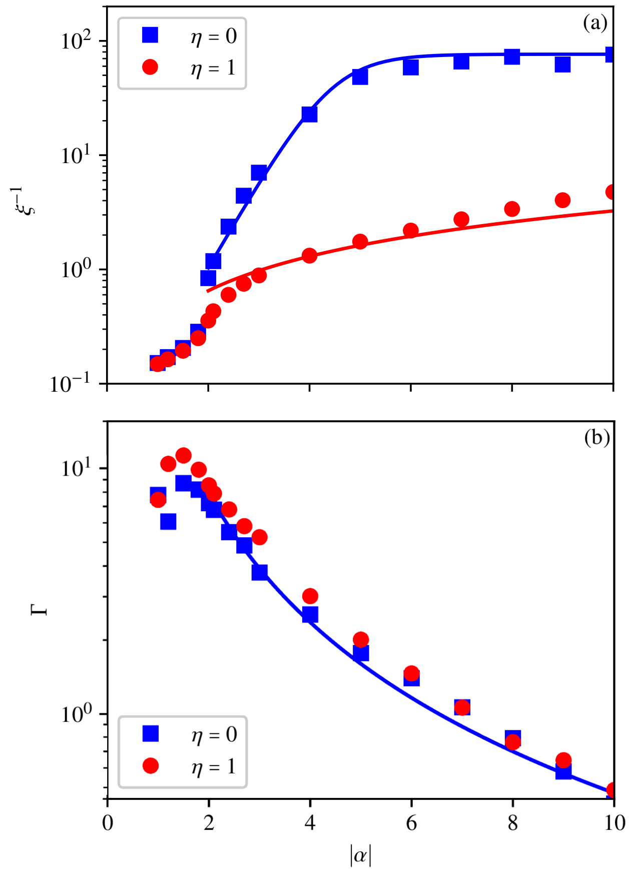

FIG. 4. Dependence of ξ^{-1} and Γ on the methylation energy parameter α. The dependence of the adaptation accuracy ξ^{-1} and the response gain Γ on the methylation energy parameter $|\alpha|$ for the cases of purely sequential ($\eta = 0$, blue symbols) and purely random ($\eta = 1$, red symbols) methylation. (a) Adaptation accuracy versus $|\alpha|$. (b) Response gain versus $|\alpha|$. The lines are guides to the eye.

state approximation, that

$$[SE] = \min([S]_0, [E]_0) \ . \tag{23}$$

With $[E]_0 < [S]_0$, the reaction speed, $v = k_{cat}[SE] = k_{cat}[E_0]$, becomes independent of the number of methylated sites, i.e., the perfect adaptation condition is satisfied.

Though this model is a mathematical possibility, it predicts a binding rate of cheR to the receptor that depends on the methylation level m because CheR binds to each of the available methylation sites randomly and therefore $k_{on} \propto (M - m)$. However, this seems to be inconsistent with recent in vitro experiments [82] that measured the kinetic rates of

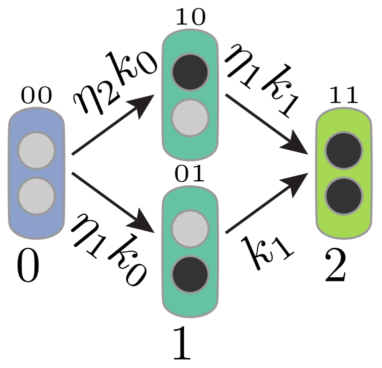

FIG. 5. Illustration of the site specific random methylation dynamics for a receptor with 2 methylation sites. The randomness parameters for the non-sequential methylation reactions can be different for the two sites: η_1 for site 1 and η_2 for site 2.

CheR (for tethered and untethered CheR) and showed the k_{on} rate to be independent of the receptor methylation level.

SUPPLEMENTARY DISCUSSION

The Che-B$^-$ mutant. Mehtylation of *E-coli* sites 1 and 2 were experimentally shown (Table I) to depend on the modification of *E-coli* site 3, that controls the sequential modification of *E-coli* site 2. Mutants lacking the methylesterase che-B, as those of Table I, are not able to demethylate the receptors and their simpler dynamics can be solved analytically. We will use the receptor with two methylation sites of Supplementary Fig. 5 to describe the dynamics of *E-coli* sites 1 and 2 of the mutants $EEDE$ and $EEQE$ of Table I. However, due to the reverse site numbering order, the numbers 1 and 2 here are the reverse of the *E-coli* receptor numbering.

The main simulation model of this paper, illustrated in Fig. 1b, has one parameter η that controls the randomness of the model. In this section we use two parameters, η_1 and η_2 in Supplementary Fig. 5, to include the possibility of distinct probabilities of random modification of sites 1 and 2 depending on the neighbor site residue. The methylation rate of μ_2 is proportional to η_2 when μ_1 is not methylated, the same factor used in the main model, $\eta_E \equiv \eta_2 \equiv \eta$. The two constants k_0 and k_1 express the possibility of different methylation rates depending on the methylation level.

Mutation of *E-coli* site 3 is equivalent to changing the value of η_1 in the model of Sup-

plementary Fig. 5. Glutamine residue in *E-coli* site 3 implies $\eta_1 = \eta_Q = 1$ in Supplementary Fig. 5, that promote sequential methylation of site 1 and leads to $p_1(t) > p_2(t)$ (EEQE with 1 and 2 reversed in Table I). Aspartate residue in *E-coli* site 3 implies $\eta_1 = \eta_D$, that inhibits sequential methylation of site 1 and leads to $p_1(t) < p_2(t)$ (EEDE with 1 and 2 reversed in Table I). We assume $\eta_Q = 1$ and, by comparing the μ_2 methylation rate of *EEQE* and *EEDE* in Table I, we concluded that $\eta_D = \eta_1 = 0.017/0.74 = 0.023$.

The distribution of the receptors among the four methylation states of Supplementary Fig. 5 obeys

$$\frac{dP_\mu}{dt} = TP_\mu \,. \tag{24}$$

with

$$P = \begin{bmatrix} P_{00} \\ P_{01} \\ P_{10} \\ P_{11} \end{bmatrix} \text{ and } T = \begin{bmatrix} -(\eta_1+\eta_2)k_0 & 0 & 0 & 0 \\ \eta_1 k_0 & -k_1 & 0 & 0 \\ \eta_2 k_0 & 0 & -\eta_1 k_1 & 0 \\ 0 & k_1 & \eta_1 k_1 & 0 \end{bmatrix} \tag{25}$$

whose solution is

$$P_{00} = e^{-(\eta_1+\eta_2)k_0 t} \tag{26}$$

$$P_{01} = \frac{\eta_1 k_0}{k_1 - (\eta_1+\eta_2)k_0}\left[e^{-(\eta_1+\eta_2)k_0 t} - e^{-k_1 t}\right] \tag{27}$$

$$P_{10} = \frac{\eta_2 k_0}{\eta_1 k_1 - (\eta_1+\eta_2)k_0}\left[e^{-(\eta_1+\eta_2)k_0 t} - e^{-\eta_1 k_1 t}\right] \tag{28}$$

$$P_{11} = \frac{k_1 B_{01} + \eta_1 k_1 B_{10}}{(\eta_1+\eta_2)k_0}\left[1 - e^{-(\eta_1+\eta_2)k_0 t}\right] - B_{10}(1 - e^{-k_1 t}) - B_{10}(1 - e^{-\eta_1 k_1 t}) \,. \tag{29}$$

With these expressions, the ratio between the methylation level of sites 1 and 2 can be calculated as

$$\frac{p_1(t)}{p_2(t)} = \frac{P_{01} + P_{11}}{P_{10} + P_{11}} \,. \tag{30}$$

The ratio of $p_1(t)/p_2(t)$ as a function of time is shown in Supplementary Fig. 6a for different values of η_1 and η_2. The minimum value of the ratio is plotted in Supplementary Fig. 6b as a function of η_1 for the rates of η_2/η_1 shown in the legend. Simulation with the MC model of the main text are shown as cyan circles, and perfectly agree with the present analytic model with $\eta_1 = \eta_2$.

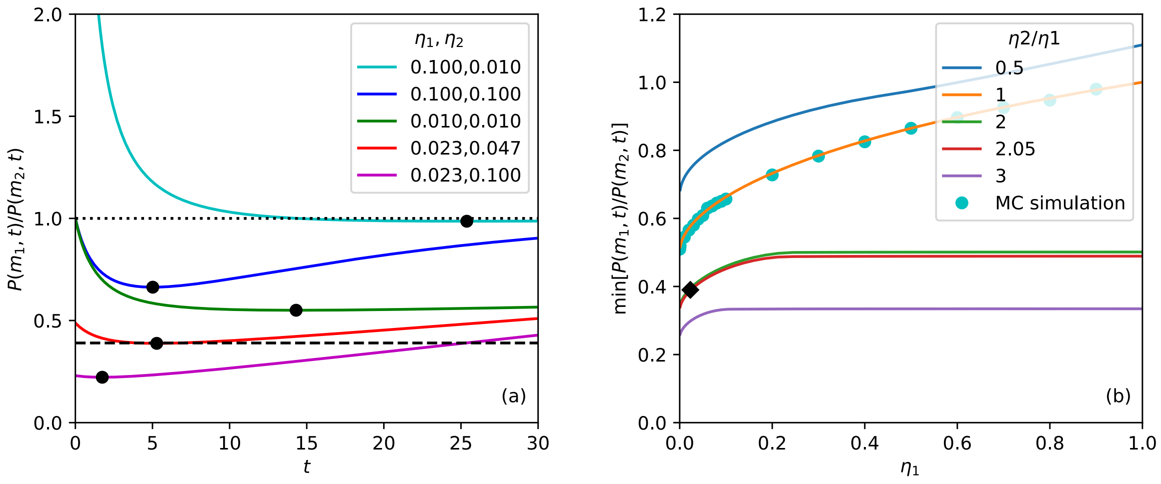

FIG. 6. Ratio of methylation rates for site 1 and site 2. (a) Ratio $p_1(t)/p_2(t)$ calculated by Supplementary Eqs. (26-30) with the values of η_1 and η_2 indicated in the legend. The black circles are the points of minimum. The black dashed line is the ratio 0.39. (b) Minimum values of the ratio $p_1(t)/p_2(t)$ as a function of η_1 for the ratios of η_2/η_1 shown in the legend. The black diamond is the point $\eta_1 = 0.023$, $\min[p_1(t)/p_2(t)] = 0.39$. The subscripts 1 and 2 used here are in the reverse of the ones in the Tar receptors.

The presence, on these equations, of distinct parameters η_1 and η_2 for random methylation of different sites, can be used to obtain more general results than those obtained from our MC simulation, where $\eta_1 = \eta_2$. For example, the ratio $p_1(t)/p_2(t) < 0.5$ requires $\eta_2 > \eta_1$, as can be seen in Supplementary Fig. 6b.

The ratio $p_1(t)/p_2(t) = 0.017/0.044 = 0.39$ experimentally obtained for *EEDE* in Table I is the black dashed line of Supplementary Fig. 6a. This is the minimum value of $p_1(t)/p_2(t)$ when $\eta_1 = \eta_D = 0.023$ and $\eta_2 = 0.047$, plotted as the red curve in Supplementary Fig. 6a. This value of η_2 sets the lower bound for the parameter η used in the model of the main text: $\eta > 0.047$. In our simulations of *E-coli* methylation dynamics we used $\eta = 0.1$, the purple line of Supplementary Fig. 6a.

Testable predictions – Adapted activity and adaptation time. We first study the effects of mutating site 1 or site 3 to be permanently unmethylated by fixing either $\mu_3 = 0$ or $\mu_1 = 0$ in our model and compare the results to those of the wt receptor where all three sites are modifiable. Note for this study, we fix $\tilde{\mu}_4 = 0$ for convenience as methylation

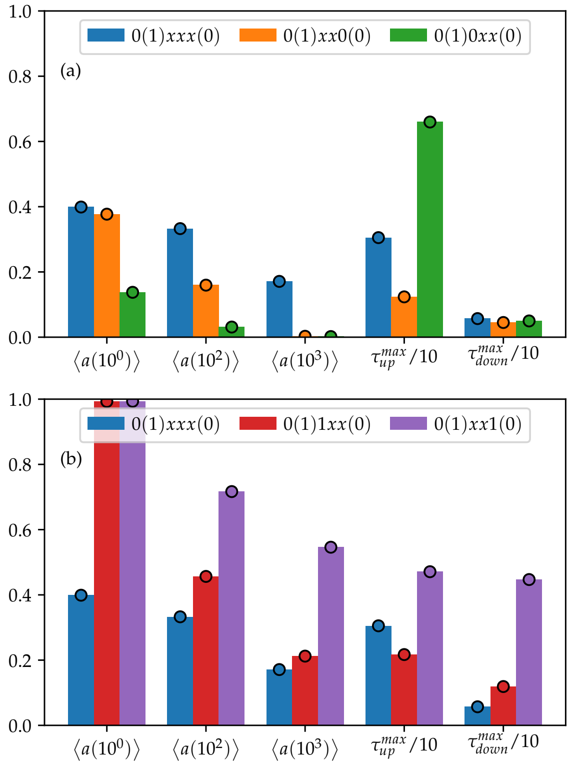

FIG. 7. Adapted activity and adaptation time for different mutant strains defined in the legends. (a) Mutants with either fixed $\tilde{\mu}_1 = 0$ (orange bars) or fixed $\tilde{\mu}_3 = 0$ (green bars) in comparison with the wt receptor (blue bars) with both sites modifiable. (b) Mutants with either fixed $\tilde{\mu}_1 = 1$ (purple bars) or fixed $\tilde{\mu}_3 = 1$ (red bars) in comparison with the wt receptor (blue bars) same as in (a). The adapted activities were shown for three concentrations $[L] = 10^0, 10^2, 10^3$ μM. The $\tau_{\rm up}^{\rm max}$ and $\tau_{\rm down}^{\rm max}$ are the maximum adaptation times for the up and down steps, respectively.

rate of site 4 is low. As shown in Supplementary Fig. 7(a), the $\tilde{\mu}_3 = 0$ mutant (0(1)0xx(0), the green bars) has the most deficient adaptation behavior with much lower adapted activities and a much slower adaptation time in response to a large increase in attractant concentration. In comparison, the effects of fixing $\tilde{\mu}_1 = 0$ (0(1)xx0(0), the orange bars) are much milder. The reason is that mutating site 1 to be permanently unmethylated still preserves the sequential methylation of site 2 by site 3. However, mutating site 3 to be permanently unmethylated disrupts the sequential methylation of site 2. Therefore, adaptation still works in mutant $0(1)xx0(0)$ but is severely impaired in the mutant $0(1)0xx(0)$.

We next study the effects of mutating site 1 or site 3 to be permanently methylated by

fixing either $\mu_3 = 1$ or $\mu_1 = 1$ in our model. As shown in Supplementary Fig. 7(b), the $\tilde{\mu}_1 = 1$ mutant (0(1)xx1(0), the purple bars) has the most deficient adaptation behavior with much higher adapted activities and a much slower adaptation time in response to a large decrease in attractant concentration. In comparison, the effects of fixing $\tilde{\mu}_3 = 1$ (0(1)1xx(0), the red bars) are much milder. The explanation is that mutating site 3 to be permanently methylated does not affect the sequential demethylation of site 2 by site 1. However, mutating site 1 to be permanently methylated disrupts the sequential demetylation of site 2. Therefore, sequential demethylation, which is important for responding to decrease in attractant concentration remains intact in mutant $1(1)1xx(0)$, but is severely impaired in the mutant $1(1)xx1(0)$.

Indirect evidence for sequential demethylation. The Trg receptor has 5 methylation sites at positions 304, 310, 311, 318, and 500. Sites 1, 3, and 4 are 7 residues apart of each other, the same distance observed between sites 1, 2, and 3 of Tar. It is reasonable to assume that these two sets of sites are subjected to the same sequential methylation dynamics. For Trg, this means methylation follows the sequence $4 \to 3 \to 1$. In [83, 84] Trg receptors are mutated to have the residues $EEQQQ$ at the aforementioned positions. Fig. 7d in [83] shows that residue 304 is quickly methylated in non-deamidated receptors. This is the expected behavior since site 3 (residue 311) is methylated and residue 304 is the next in the methylation sequence.

If we assume that deamidation and demethylation by CheB follow the same sequence $1 \to 3 \to 4$, the deamidation (by CheB) of a $EEQQQ$ Trg receptor would happen preferentially at position 311 as site 1 (residue 304) is unmethylated. Furthermore, if residue 311 is deamidated, this residue (311) would be the preferred residue for methylation (by CheR) because site 4 (residue 318) is methylated, and methylation of glutamate at 304 would be much slower. This is exactly what was observed in Fig. 7c of [83], which agrees with the predictions from sequential deamidation/demethylation.

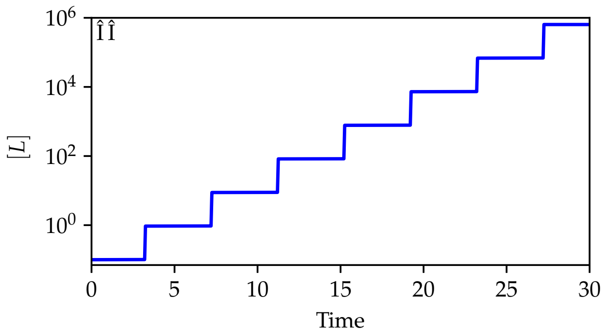

FIG. 8. Time series of the step changes in external stimulus (ligand concentration $[L]$) used in the simulations for Fig. 2 in the main text.
